# MEK/CDK4,6 co-targeting is effective in a subset of NRAS, BRAF and ‘wild type’ melanomas

**DOI:** 10.18632/oncotarget.26204

**Published:** 2018-10-09

**Authors:** Christian Posch, Martina Sanlorenzo, Jeffrey Ma, Sarasa T. Kim, Mitchell Zekhtser, Susana Ortiz-Urda

**Affiliations:** ^1^ Technical University of Munich, Department of Dermatology and Allergy, 80802 Munich, Germany; ^2^ University of California San Francisco, Department of Dermatology, Mt. Zion Cancer Research Center, 94115 San Francisco, USA; ^3^ Faculty of Medicine, Sigmund Freud University, 1020, Vienna, Austria; ^4^ Institute of Cancer Research, Medical University of Vienna, 1090 Vienna, Austria

**Keywords:** NRAS, CDK4, BRAF, MEK, melanoma

## Abstract

Targeted therapy has become a cornerstone for the treatment of melanoma patients. Targeting NRAS function is particularly challenging. To date, only single MEK inhibitor treatment was able to show minimal clinical efficacy. The discovery that co-targeting of MEK and CDK4,6 has antitumor activity created excitement for patients and clinicians; however, it is largely unknown if only NRAS mutant patients might benefit from MEK/CDK4,6 blockade.

In this study we investigate response patterns of NRAS, BRAF mutant and ‘wild type’ melanoma cells *in vitro* and *in vivo* when challenged with inhibitors of MEK, CDK4,6 and the combination of both. Data revealed, that *in vitro* growth response patterns of cells treated with the MEK/CDK4,6 combination correspond to *in vivo* efficacy of MEK/CDK4,6 co-targeting in melanoma xenograft models. Strikingly, this was consistently observed in NRAS and BRAF mutant, as well as in ‘wild type’ melanoma cells. Additionally, cells displaying elevated p-Rb levels after single MEK inhibition, showed more effective growth reduction with MEK/CDK4,6 co-targeting compared to single MEK inhibitor treatment *in vivo*. Findings indicate that combined MEK/CDK4,6 inhibition could offer an effectively therapeutic modality in a subset of BRAF and NRAS mutant, as well as ‘wild type’ melanoma patients.

## INTRODUCTION

Despite recent advances, treatment of metastatic melanoma remains challenging. Inhibitors specifically targeting mutant kinases such as KIT and BRAF as well as new antibody-mediated immunotherapies have improved progression free survival (PFS) and overall survival (OS). To date, treatment decisions are based on genetic testing of mutations in a limited number of oncogenes. This approach has significantly improved patient-selection for successful kinase inhibitor treatment [1-4]. Thus, the detection of mutations in recognized oncogenes is an important tool to improve the efficacy of targeted inhibitor treatment. However, these tests only provide a very limited view on the genetic background of a given malignancy and seem to be insufficient to be stand-alone predictors for an effective and personalized choice of targeted inhibitors or their combinations. An extensive body of literature has been published trying to define genetic tumor signatures for the prediction of treatment outcome with varying success [5-9]. Such strategies have the potential to not only improve our understanding of tumor biology, but might also help to refined selection of patients that are most likely to respond to the respective treatment modalities.

Treatment of (N)RAS mutant tumors is particularly challenging. To date, it has been impossible to directly target mutant RAS in a clinically meaningful way and therapeutics including chemotherapy, radiation therapy and single targeted therapy using MEK inhibitors only show very limited activity once a patient has failed immunotherapy. Thus, recent findings describing that a MEK/CDK4,6 inhibitor combination is mimicking NRAS extinction in melanoma cells created excitement for physicians and patients [10]. These results were followed by the initiation of first clinical trials, however, so far results are not meeting pre-clinical expectations with only a small number of NRAS mutant melanoma patients responding to MEK/CDK4,6 inhibition [11]. We followed up on this discovery in an attempt to improving the stratification of NRAS mutant melanoma patients that are most likely to respond. Additionally, we included BRAF, GNAQ and KIT mutant melanoma cells in our analyses. Data revealed that *in vitro* growth response patterns using a MEK/CDK4,6 combination correlate with a response to the MEK/CDK4,6 combinatorial treatment *in vivo*. Additionally, induction of the cell cycle regulator p-Rb after single MEK inhibitor was found in tumors with effective co-targeting of MEK/CDK4,6 *in vivo*. Strikingly, results of this study reveal that this finding holds true for NRAS and BRAF mutant, as well as ‘wild type’ melanoma cells.

## RESULTS

### Reduction of cell viability in a subset of NRAS mutant melanoma cells with MEK/CDK4,6 inhibition *in vitro*

**Figure 1:**
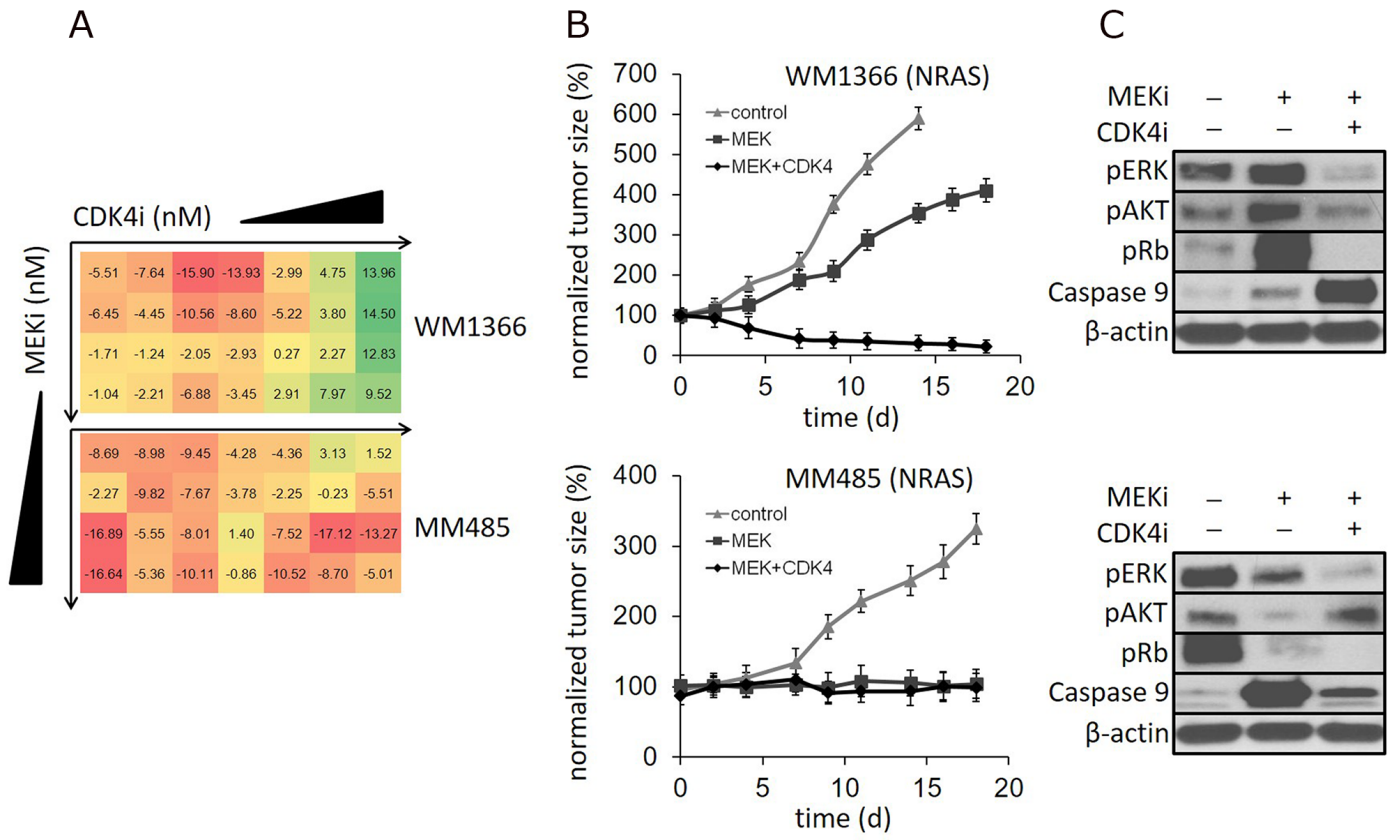
**(A)** NRAS mutant melanoma cell lines WM1366 and MM485 incubated with increasing concentrations of a MEK and CDK4,6 inhibitor in combination (MEKi: 1nM-125nM; CDK4,6i: 0.04nM-625nM). The numbers represent the relative change in viability compared to single MEK inhibitor treatment. (Color codes: linear range from ‘red’ - representing less reduction in cell viability by MEK/CDK4,6 compared to single MEK inhibition - to ‘green’ - representing increased reduction of cell viability by MEK/CDK4,6 compared to single MEK inhibition). **(B)** NRAS mutant human melanoma xenografts in mice treated with vehicle control, a MEK inhibitor or the MEK/CDK4,6 inhibitor combination: Tumor size reduction with MEK/CDK4,6 compared to single MEK inhibition of WM1366 tumors, but not of MM485 tumors. **(C)** Respective immunoblots of tumor tissue: Induction of p-Rb by single MEK inhibitor treatment in WM1366 tumors. In contrast, p-Rb reduction by single MEK inhibition in MM485 tumors. (^*^mice had to be euthanized due to tumor size, N=4).

In order to investigate the effects on cell viability using the MEK/CDK4,6 inhibitor combination, we evaluated the response of 10 NRAS mutant melanoma cell lines to the MEK inhibitor GSK1120212 (trametinib), the CDK4,6 inhibitor PD0332991 (palbociclib) and the combination of both inhibitors *in vitro*. Supplementary Table 1 details mutations of all cell lines used in this study. All NRAS mutant cells were sensitive to the MEK inhibitor at concentrations ranging from 1nM to 125nM resulting in varying degrees of growth reduction. None of the NRAS mutant melanoma cells showed a reduction of viability in response to the CDK4,6 inhibitor at concentrations used in this study (Supplementary Figure 1). In several cell lines including WM3060, WM3629, WM3670 and MM415 we observed a significant growth advantage of cells treated with PD0332991 compared to vehicle treated controls. The combination of the MEK and the CDK4,6 inhibitor effectively reduced cell viability in WM1366, D04, WM3629, WM3060, MaMel27II, MaMel30I and Sk-Mel-2 cells *in vitro*. In MM485, WM3670 and MM415 cells the addition of the CDK4,6 inhibitor had no beneficial effect in reducing cell growth or even antagonized the effects of MEK inhibition *in vitro* (Figure 1A, Supplementary Figure 2A, and 3A).

### Select NRAS mutant cells are sensitive to MEK/CDK4,6 inhibition *in vivo*

Next we sought to determine if the differential *in vitro* sensitivity to MEK/CDK4,6 inhibition correlates with *in vivo* treatment response. Thus, we established human melanoma xenograft models using WM1366, MM415, MM485, WM3629, D04 and WM3670 cells. Reflecting current clinical treatment modalities in patients treated with small molecule inhibitors, mice received either the MEK inhibitor alone or the MEK/CDK4,6 combination. Due to the lack of activity of the CDK4,6 inhibitor *in vitro*, single CDK4,6i treatment was not further assessed *in vivo*. Control cohorts received the vehicle only. Treatment was administered by oral gavage, with the MEK inhibitor at a dosage of 2mg/kg/day and the CDK4,6 inhibitor at a dosage of 150mg/kg/day. Treatment was initiated when the tumor reached a size of about 100mm^3^. The MEKi alone stabilized the growth of D04, MM415, WM3670 and MM485 tumors in mice, whereas WM3629 and WM1366 tumors only showed a slight reduction of tumor size compared to mice treated with vehicle control. The combination of the MEK and CDK4,6 inhibitor was effective in mice bearing WM1366, D04 and WM3629 tumors, abolishing WM1366 tumors, significantly decreasing the size of D04 tumors and stabilizing the size of WM3629 tumors. In mice bearing MM415, MM485 and WM3670 tumors, the MEK/CDK4,6 combination did not further decrease the size of the xenograft compared to MEK inhibition alone (Figure 1B, Supplementary Figure 2B and 3A). Altogether, *in vivo* response rates are in line with the observed sensitivity of the cell lines *in vitro* and reveal that select NRAS lines can effectively be decreased in size with the MEK/CDK4,6 combination.

### Increasing levels of p-Rb in response to MEK inhibition are indicative for effective MEK/CDK4,6 combinatorial therapy

To determine signaling changes in WM1366 and MM485 tumors derived from xenografted mice treated with the respective inhibitor(s) or the vehicle control, we extracted total protein from tumor tissue of the respective treatment groups at the end of a 3-weeks treatment cycle. Immunoblot analyses revealed largely unchanged or reduced p-ERK protein levels after MEK inhibition in WM1366 and MM485 tumors, respectively. We also noticed an induction of p-AKT in WM1366 tumors treated with the MEKi only. Interestingly, the common cell cycle downstream target, pRb, was strongly induced by the MEKi in WM1366 tumors whereas a marked reduction was noticed in MM485 tumors. The reduction in pRb in MM485 tumors was in lieu to marked induction in the pro-apoptotic marker caspase 9, whereas only a slight increase in caspase 9 was noticed in WM1366 tumors after MEK inhibition. WM1366 tumors in the mice receiving the MEK/CDK4,6 combination revealed abolished pRb levels and strong induction of caspase 9 (Figure 1C). In MM485 tumors pRb was also further reduced by the MEK/CDK4,6 combination, however, caspase 9 levels were markedly lower than with MEKi treatment alone. Changes in the pro-apoptotic marker caspase 9 are in line with the observed tumor size reduction in mice bearing WM1366 tumors using the combinatorial treatment. The same treatment response and signaling patterns were observed in xenograft models using D04 and WM3670 cells. D04 tumors showing induction of p-Rb after MEKi treatment could further be reduced in size with the addition of the CDK4,6i. In contrast, WM3670 tumors responding with decreased p-Rb protein levels after MEKi treatment did not shrink with the MEK/CDK4,6 combination (Supplementary Figure 2C). Results suggest that the signaling changes in the cell cycle regulator Rb in response to single MEKi treatment can be used to predict the efficacy of the MEK/CDK4,6 combination.

### Effective growth inhibition in BRAF mutant and ‘wild-type’ melanoma cell lines with a MEK/CDK4,6 combination

Next, we assessed if the observed *in vitro* growth response and signaling patterns in human NRAS mutant lines might also predict MEK/CDK4,6 inhibitor sensitivity of cells with different driving mutations. We incubated the human BRAF mutant cell lines A2028, A375, MM466 and Sk-Mel-28, the GNAQ mutant cell lines Mel202 and Omm1.3, the KIT mutant line WM3211, as well as C918 cells that do not harbor mutations in NRAS, BRAF, GNAQ/GNA11 and KIT (called ‘wild type’ in the following) with the MEKi, CDK4,6i or the combination of both (Supplementary Figure 3B and 4). Similar to findings in NRAS mutant lines, single CDK4,6 inhibition did not decrease cell viability in any of the cells tested using the concentrations used in this study, whereas MEK inhibition reduced cell viability in all cell lines at varying degrees using MEKi concentrations of 1nM to 125nM. Comparing the results of single MEK inhibition with the MEK/CDK4,6 combination, data revealed that cell viability could further be reduced by the inhibitor combination in select cell lines. The combination effectively reduced viability in the BRAF mutant lines MM466 and Sk-Mel-28, the KIT mutant line WM3211, the ‘wild-type’ line C918 and, to an even greater extent, in both GNAQ mutant lines Mel202 and OMM1.3. Modest reduction of viability was found in A375 cells. In A2028 cells co-targeting of CDK4,6 did not further reduce viability compared to MEK inhibition alone.

The results of a xenograft model of human melanoma using the MM466 and C918 cells were in line with *in vitro* findings and showed reduced growth of tumors in the combinatorial treatment groups compared to MEK inhibition alone or vehicle controls (Supplementary Figure 3B).

## DISCUSSION

The development of specific small molecules revolutionized melanoma therapy. This is particularly true for BRAF(V600) mutant disease and to a lesser extent for KIT mutant melanoma [1, 2, 4, 5]. Yet, there is a significant number of patients failing therapy. Targeted treatment of patients with NRAS mutant melanoma, remains an unsolved challenge and current therapeutic modalities only barely improve overall survival. To date, immunotherapy and targeted inhibition of NRAS downstream signaling mediators are the most promising strategies. In this study we first investigated a promising new combinatorial targeted therapy (MEK/CDK4,6 blockade) for NRAS mutant melanoma in a large set of NRAS mutant cell lines. Even though all cells were NRAS mutant, results showed varying sensitivity to MEK/CDK4,6 inhibition *in vitro* and *in vivo*: Induction of the central cell cycle regulator Rb after single MEK inhibition was indicative of effective MEK/CDK4,6 inhibition *in vivo*. Additionally, *in vivo* responses echoed *in vitro* growth reduction using the MEK/CDK4,6 combination. Second, we expanded our cell line panel and included BRAF, and ‘wild type’ melanoma cells including GNAQ and c-KIT mutants. Similar to findings in NRAS mutant cells, also a subset of melanoma cells with different genetic driver mutations could effectively be blocked with MEK/CDK4,6 inhibition. Again, *in vitro* response was similar to *in vivo* findings suggesting that select melanoma cells, regardless of their driver mutation are sensitive to MEK/CDK4,6 blockade.

We used a MEK inhibitor as a backbone for combinatorial targeted regimens for several reasons. MEK is a central element of the MAPK signaling cascade, which is believed to be one of the most important pathways for the survival and maintenance of melanoma cells. Blocking of MAPK signaling using specific small molecule inhibitors of BRAF and MEK are central in the treatment of BRAF mutant melanoma patients. Single MEK blockade also revealed clinical activity in NRAS activated melanoma patients [12]. However, even in the small subset of NRAS mutant patients that initially responded to MEK inhibition rapid development of resistance to therapy was noticed. The activation of pro-survival signaling cascades such as the PI3K/mTOR pathway, restored MAPK signaling or the induction of cell cycle mediators are believed to limit the clinical activity of single MEK inhibitor treatment [13, 14]. Thus, current research focuses on the discovery of NRAS co-extinction-targets to MEK inhibition. First preclinical results show that NRAS mutant tumors can be selectively inhibited by combined MEK/PI3KmTOR, MEK/metformin and MEK/CDK4,6 inhibition [10, 14, 15]. The latter combination is, to date, the most advanced therapeutic modality with several clinical trials currently recruiting patients. Indeed, first pre-clinical results created excitement that combined MEK/CDK4,6 might serve as a therapy for indirect NRAS targeting. Yet, increasing evidence suggests that NRAS mutations alone do not predict efficacy of MEK/CDK4,6 blockade [11, 16-18]. Additionally, the inducible NRAS mutant melanoma model used in the discovery of the MEK/CDK4,6 combination was performed in CDKN2A^null^ mice [10]. CDKN2A is a critical regulator of CDK4 and thus the cell cycle. It is likely that the preexisting alterations of the cell cycle in this mouse model affected the observations and conclusions of the study. Second, it is important to consider that the database derived observation that CDK4 is a MEK co-extinction target mimicking NRAS abrogation is, at least in part, affected by the frequent cell cycle alterations in melanoma (models) in general [19]. With this in mind, a conclusion that specifically mutant NRAS renders cells sensitive to a MEK/CDK4,6 inhibitor combination raises concerns. In our hands, only a subset of NRAS mutant melanomas responds to MEK/CDK4,6 inhibition. This might subsequently limit the clinical efficacy of this inhibitor combination mainly due to a suboptimal selection of patients that are currently believed to benefit from these drugs.

Mutant NRAS signals through a multitude of downstream cascades and it appears that the evaluation of signaling events in response to MEK inhibition could serve helpful in predicting treatment efficacy using combinatorial treatment modalities also in the clinic. Such cell-, and patient-specific response patterns could be used to optimize the selection of combinatorial regimens. This might hold true not only for NRAS mutant cells, but also other melanoma cells with different genetic characteristics.

Activation of the cell cycle pathway in response to MEK inhibition, evidenced by the increase of downstream effector proteins such as p-Rb, was found in cells with increased cell death and tumor shrinkage when MEK and CDK4,6 inhibitors were used. Without an increase of p-Rb after MEK inhibition, the addition of the CDK4,6 inhibitor did not further reduce tumor size. Findings support that not the NRAS mutation alone renders cells sensitive to MEK/CDK4,6 inhibition, but the involvement of the cell cycle pathway and its modulation upon MEK targeting. Our results do not disparage the achievements of previous studies, but much rather add additional insights that might help to better stratify and identify patients that are more likely to respond to combined MEK/CDK4,6 inhibition.

## MATERIALS AND METHODS

### Cell culture

Human melanoma cell lines WM3629, WM3670, WM3060 and WM1366 were obtained from the Coriell Institute; cell lines D04, Sk-Mel-2, MM485, MM415, MaMel27II, A375, A2058, Sk-Mel28, MM466, and MaMel30I were a generous gift from Boris Bastian at the University of California, San Francisco. Cell lines WM3629, WM3670, WM3060, and WM1366 were maintained in MCDB153 media supplemented with 20% Leibovitz’s L-15 Media, 2% FBS and 1.68 mM CaCl2, while cell lines D04, MM485, MM415, MaMel27II, MaMel30I, A375, A2058, Sk-Mel28, and MM466 were maintained in RPMI-1640 media supplemented with 10% FBS. All cell lines were incubated at 37°C under 5% CO_2_. Mutation statuses of each cell line pertaining to this study are listed in Supplementary Table 1.

### Inhibitor and cell viability assays

All inhibitors used in the study were purchased from Selleck Chemicals and ChemieTek. Cell viability assays were performed in 96 well plates with approximately 5000 cells per well. Inhibitors were added to cell lines 24 hours after plating of cells and incubated for 72 hours. Viability assays were tested at least in duplicates. The relative number of viable cells was calculated using CellTiter-Glo® (Promega, G7570). Total luminescence was measured on the SynergyHT plate reader (BioTek) using Gen5 software (Version 1.11.5). Cells were treated with inhibitors at a dose range of 0.04nM to 125nM for the inhibitor GSK1120212 and 0.04 to 625nM for the inhibitor PD0332991. The relative change in cell viability was calculated in MS Excel. The effect size comparing MEK and MEK+CDK4,6 inhibition is displayed as the numerical relative change in cell viability between the two treatment conditions. Additionally, values are color-coded ranging from green (pronounced reduction of cell viability of MEK+CDK4,6 compared to single MEK inhibition) to yellow (similar effect of MEK+CDK4,6 and single MEK inhibition) to red (more reduction of cell viability by single MEK inhibition compared to MEK+CDK4,6 inhibition).

### Immunoblots

Cell lines were plated in 6-well plates 24h hours before incubation with the inhibitors. Cells were washed with 1x PBS and lysed using radioimmunoprecipitation (RIPA) buffer [150 mM NaCl, 1% (vol/vol) Nonidet P-40, 0.5% (wt/vol) sodium deoxycholate, 0.1% (wt/vol) SDS] in 50 mM Tris-HCl (pH 8.0) supplemented with protease and phosphatase inhibitors (78442; Pierce) according to the manufacturer’s protocol. Protein concentrations were determined using the BCA Protein Assay kit (23235; Pierce). Total protein in 1x Laemmli buffer with 10% 2-mercaptoethanol was separated by SDS-PAGE, transferred 1 h to a PVDF membrane (IPVH00010; Millipore) by electro-blotting with 20% (vol/vol) methanol and blocked for 1 h in 5% (wt/vol) dry milk/TBS/0.1% (vol/vol) Tween-20. Membranes were incubated overnight at 4°C with primary antiserum following incubation with horseradish peroxidase-conjugated secondary antiserum for 1 h and developed using enhanced chemiluminescence (32105; Pierce or 64201BP; Millipore). Phospho-ERK, phospho-AKT, cleaved caspase 9, and phospho-Rb antibodies were purchased from Cell Signaling Technologies. Beta-actin antibodies were purchased from Sigma.

### Mouse xenografts

A total of 5-8^*^10^6^ cells of each cell line was used for subcutaneous injection in CrTac:NCr-Foxn1nu mice. Tumor size was calculated using the equation for a triaxial ellipsoid. Inhibitor treatment was initiated when tumors reached a volume of 80-100mm^3^. Inhibitor concentrations for single and combination therapy used *in vivo* were 2mg/kg/day for GSK1120212 and 150mg/kg/week for PD0332991. Compounds were administered by oral gavage fife times a week over a period of three weeks. Tumor size changes, body weight and clinical evaluations were carried out three times per week. All animal studies were approved by IACUC/LARC of the University of California San Francisco (AN086990).

## SUPPLEMENTARY MATERIALS FIGURES AND TABLE


