# Supplementary material for: MEK/CDK4,6 co-targeting is effective in a subset of NRAS, BRAF and ‘wild type’ melanomas
Source: Oncotarget. 2018 Oct 9;9(79):34990–5. doi: 10.18632/oncotarget.26204 (PMC6201855; doi:10.18632/oncotarget.26204)
Supplement: Supplementary file 1 [file oncotarget-09-34990-s001.pdf]

MEK/CDK4,6 co-targeting is effective in a subset of NRAS, BRAF and 'wild type' melanomas

SUPPLEMENTARY MATERIALS

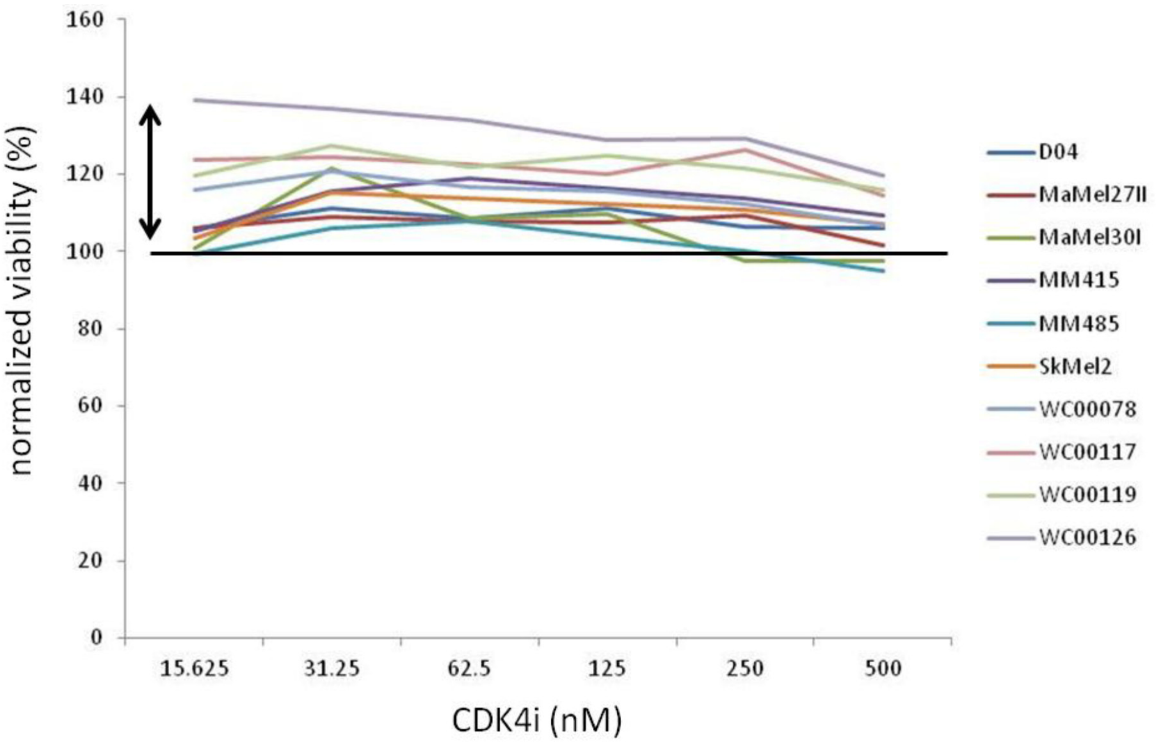

Supplementary Figure 1: Ten NRAS mutant human melanoma cell lines were incubated with the CDK4/6 inhibitor PD0332991 (palbociclib). No reduction of cell viability was detected with the concentrations used (15.6nM-500nM).

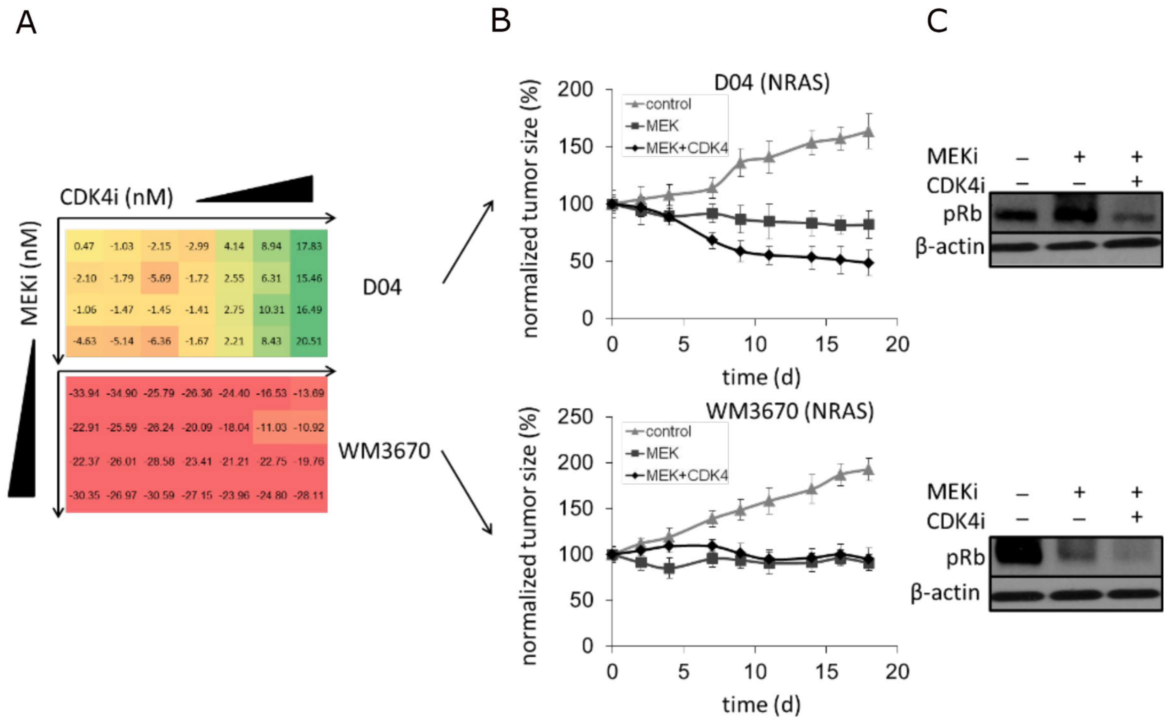

Supplementary Figure 2: **(A)** D04 and WM3670 NRAS mutant melanoma cell lines were incubated with increasing concentrations of a MEK and CDK4 inhibitor (MEKi: 1nM-125nM; CDK4,6i: 0.04nM-625nM). The numbers represent the relative change in viability compared to MEK inhibitor treatment alone. (Color codes: linear range from 'red' - representing less reduction in cell viability by MEK/CDK4,6 compared to single MEK inhibition - to 'green' - representing increased reduction of cell viability by MEK/CDK4,6 compared to single MEK inhibition). **(B)** Growth curves of D04 and WM3670 NRAS mutant human melanoma xenografts in mice treated with vehicle control, a MEK inhibitor or the MEK/CDK4 inhibitor combination. **(C)** Respective immunoblots of tumor tissue show an induction of p-Rb in D04 cells and reduction of p-Rb in WM3670 cells after MEK inhibitor treatment. (N=4).

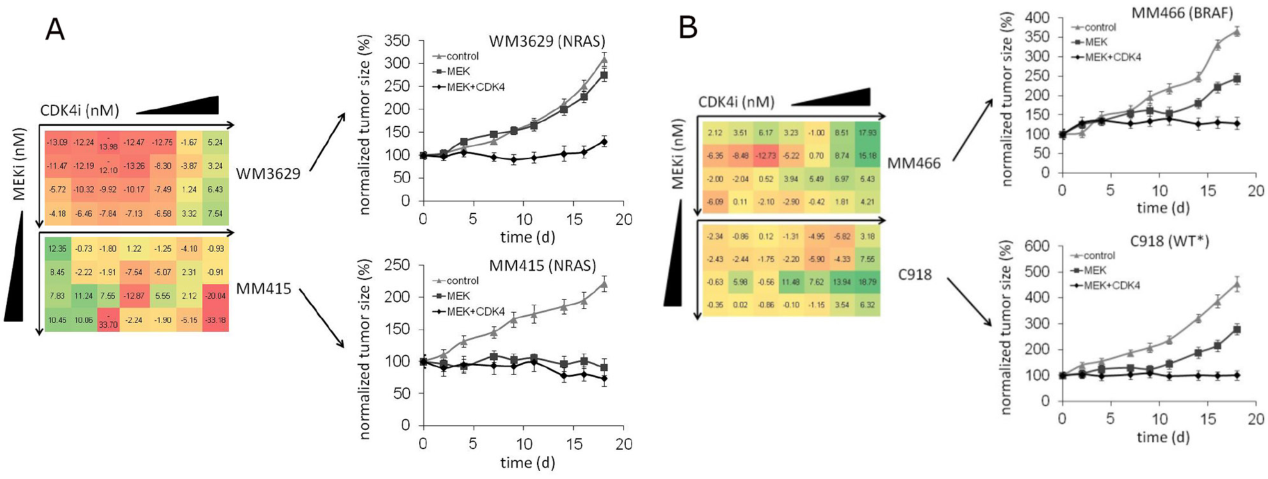

Supplementary Figure 3: **(A)** WM3629 and MM415 NRAS mutant melanoma cell lines were incubated with increasing concentrations of a MEK and CDK4 inhibitor (MEKi: 1nM-125nM; CDK4,6i: 0.04nM-625nM). The numbers represent the relative change in viability compared to MEK inhibitor treatment alone. (Color codes: linear range from 'red' - representing less reduction in cell viability by MEK/CDK4,6 compared to single MEK inhibition - to 'green' - representing increased reduction of cell viability by MEK/CDK4,6 compared to single MEK inhibition). Corresponding growth curves of WM3629 and MM415 xenografted tumors. (N=4). **(B)** The BRAF(V600E) mutant lines MM466 and the 'wild type' cell lines C918 were incubated with increasing concentrations of a MEK and CDK4 inhibitor. The numbers represent the relative change in viability compared to MEK inhibitor treatment alone. (Color codes: continuous range from 'red' representing antagonism to 'green' representing synergism). Corresponding growth curves of MM466 and C918 xenografted tumors. (N=4).

Supplementary Table 1: Mutation status of cell lines used in this study

Cell line	mutation
D04	NRAS(Q61L)
MM415	NRAS(Q61L)
MM485	NRAS(Q61R)
WM1366	NRAS(Q61L)
Sk-Mel-2	NRAS(Q61K)
WM3060	NRAS(Q61K)
MaMel27II	NRAS(G12D)
MaMel30I	NRAS(G13D) BRAF(D594N)
WM3629	NRAS(G12D) BRAF(D549G)
WM3670	NRAS(G12D) BRAF(G469E)
Ma-Mel-144aI	KIT(S476I)
WM3211	KIT(L576P)
Sk-Mel-28	BRAF(V600E)
MM466	BRAF(V600E)
C918	WT
Mel202	GNAQ(Q209L)
OMM1.3	GNAQ(Q209P)

(WT: wild type for BRAF, NRAS, KIT and GNAQ/GNA11 hotspot mutations).

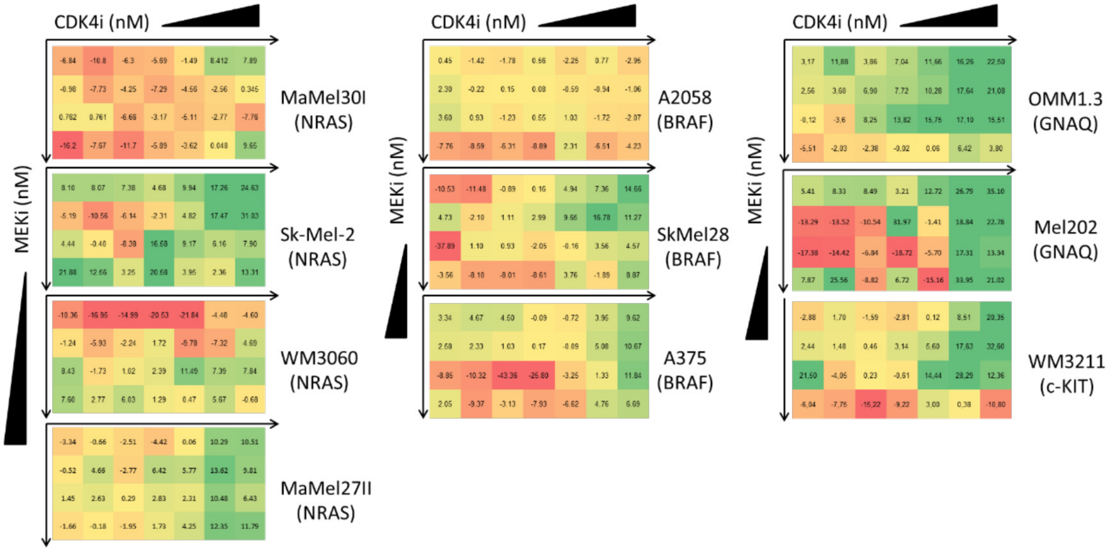

Supplementary Figure 4: *In vitro* growth response results of 4 additional human NRAS mutant melanoma cell lines (MaMel30I, Sk-Mel-2, WM3060, MaMel27II) and the BRAF(V600E) mutant lines A2058, SkMel28 and A375, as well as the GNAQ mutant line Mel202, and OMM1.3 and the c-KIT mutant line WM3211 (MEKi: 1nM-125nM; CDK4,6i: 0.04nM-625nM). The numbers represent the relative change in viability compared to MEK inhibitor treatment alone. (Color codes: linear range from 'red' - representing less reduction in cell viability by MEK/CDK4,6 compared to single MEK inhibition - to 'green' - representing increased reduction of cell viability by MEK/CDK4,6 compared to single MEK inhibition).
